# Development and Validation of a Noninvasive Model of Mixed Venous Oxygen Saturation in Heart Failure

**DOI:** 10.1016/j.jacadv.2025.102484

**Published:** 2026-01-28

**Authors:** Adam K. McDiarmid, Bradley S. Chambers, David A. Broadbent, Roshan Patel, Gareth Matthews, Oscar Gonzalez-Fernandez, Sven Plein, Pankaj Garg, Peter P. Swoboda

**Affiliations:** a Freeman Hospital, Newcastle Hospitals NHS Trust, Newcastle, United Kingdom; b Biosciences Institute, Newcastle University, Newcastle, United Kingdom; c Leeds Institute of Cardiovascular and Metabolic Medicine, University of Leeds, Leeds, United Kingdom; d Leeds Teaching Hospitals, Leeds, United Kingdom; e Norwich Medical School, University of East Anglia, Norwich, United Kingdom; f Norfolk and Norwich University Teaching Hospitals, Norwich, United Kingdom

**Keywords:** heart failure, cardiac catheterization, MRI, CMR

## Abstract

**Background:**

Mixed venous oxygen saturation (SvO_2_), measured with right heart catheterization, is a crucial prognostic tool in patients with heart failure. The prognostic significance of SvO_2_ estimated noninvasively using cardiovascular magnetic resonance (CMR) from the T2 of intracardiac blood pools remains unknown.

**Objectives:**

The objective of the study was to develop a CMR model of mixed venous saturation (imaging-derived SvO_2_ [iSvO_2_]), and establish if it is associated with future adverse events in heart failure.

**Methods:**

The iSvO_2_ was modeled in the discovery cohort (N = 30), who underwent CMR T2 mapping and invasive right heart catheterization, by linear regression. The validation cohort of 628 patients with recently diagnosed heart failure underwent clinical assessment, CMR, and follow-up (median 3 years [IQR: 1.5-4.8]) for a primary endpoint of all-cause mortality or heart failure hospitalization.

**Results:**

Significant positive correlation was found between the ratio of right ventricular blood pool T2/left ventricular blood pool T2 and invasive mixed venous oxygen saturation (R = 0.82; 95% CI: 0.66-0.91; *P* < 0.001), giving the equation: iSvO_2_ = 95·(RV−T2_BP_/LV−T2_BP_). In the validation cohort, there was a strong association between iSvO_2_ and the primary endpoint (HR: 0.66 for 10% change in iSvO_2_; 95% CI: 0.54-0.81; *P* < 0.001), which remained significant after adjusting for age, sex, left ventricular ejection fraction, right ventricular ejection fraction, N-terminal pro–B-type natriuretic peptide, NYHA functional class, and diabetes.

**Conclusions:**

The CMR iSvO_2_, measured from simple T2 maps of left and right ventricular blood pool, allows accurate estimation of the invasive SvO_2_. In a real-world heart failure registry, iSvO_2_ is an independent predictor of mortality and heart failure hospitalization.

Understanding prognosis in heart failure (HF) is essential to guide treatment planning, referral decisions, and to empower patients. In clinical practice, assessment of cardiac function by echocardiography has become the de facto arbiter, with left ventricular (LV) ejection fraction (LVEF) used to grade HF severity, determine access to pharmacological and device interventions, and define response to therapy.^1^^,^^2^ As myocardial contractility falls, the left ventricle dilates, and as such, ejection fraction alone ceases to provide an accurate assessment of cardiac output. In contrast, LVEF may be preserved in restrictive cardiomyopathies, and it does not consistently predict outcomes across the broader HF population.^3^^,^^4^ Physiological measures such as cardiopulmonary exercise testing and right heart catheterization (RHC) are used in the advanced HF population to help determine suitability for advanced HF therapies.^5^

Mixed venous oxygen saturation (SvO_2_) provides a measure of tissue oxygen extraction and is measured during RHC in the main pulmonary artery. In HF, low SvO_2_ is found in low cardiac output states and reflects a relative increase in the proportion of oxygen extracted from passing red cells as global oxygen delivery falls (DO_2_).^6^ As such, in the absence of anemia, myocardial oxygen consumption (mVO_2_) may be viewed as a surrogate for cardiac index. mVO_2_ has a prognostic value, is used as a tool to monitor response to therapy, and offers physiological data to guide decision-making in advanced HF and cardiogenic shock.^7^ It is only possible to measure mVO_2_ during RHC, which can be uncomfortable, is invasive and carries a risk of complications.^8^ There is, therefore, a clinical need for an imaging biomarker to estimate mVO_2_ noninvasively.

Cardiovascular magnetic resonance (CMR) is increasingly integrated into cardiology practice, with utility in assessing cardiac structure, function, hemodynamics^9^, ^10^, ^11^, ^12^ and, importantly, tissue characterization. T2 mapping is typically used to identify and quantify myocardial edema, but is also sensitive to blood oxygen levels.^13^ This technique is based on the principle that the T2 relaxation time of blood depends on its oxygenation. Deoxygenated hemoglobin is paramagnetic, shortening transverse relaxation (T2 and T2∗), whereas oxygenated hemoglobin lengthens transverse relaxation. This phenomenon has long been recognized as a viable intrinsic contrast mechanism for magnetic resonance imaging,^14^ with the blood oxygenation level dependent contrast effect having found widespread adoption in functional magnetic resonance imaging of the brain.^15^ It has also found application for assessing oxygenation in various other body tissues including myocardium.^16^^,^^17^ This effect has been used with quantitative relaxometry techniques to estimate absolute blood oxygen saturation.^18^, ^19^, ^20^, ^21^ However, accurate quantification requires knowledge of additional blood properties (hematocrit and/or T1), nonlinear model fitting and control of acquisition parameters (such as refocusing pulse separation) that are not commonly available in product T2 mapping sequences, so such methods are challenging to apply widely in clinical settings. Studies have identified that previously ignored differences in LV and right ventricular (RV) blood T2 values may be clinically relevant, with the ratio between the right and left ventricles correlating with exercise capacity in HF and pulmonary pressures in pulmonary hypertension.^22^^,^^23^ However, currently, T2 mapping has not been validated against right heart oxygen saturation, and its prognostic relevance in HF remains undetermined.

We aimed to develop a CMR model for measuring SvO_2_ noninvasively and to test the hypothesis that this measure, termed iSvO_2_, where “i” stands for imaging-derived, is independently associated with outcomes in a cohort of well-phenotyped HF patients.

## Methods

### Ethical approval

Ethical approval for the studies was obtained through the Health Research Authority's Research Ethics Committee (Norwich: 21/NE/0149, Leeds: 17/YH/0300 and 20/NW/0326). Informed consent was obtained from all patients who were prospectively recruited. For the Newcastle discovery catheter cohort, anonymized, observational, and retrospective data were used, in keeping with local and national information governance guidelines. All research adhered to the principles outlined in the Declaration of Helsinki.

### Study population

#### Discovery cohort

In the discovery cohort, patient data were collected from Newcastle and Norwich centers. Patient data in Newcastle patients were collected from individuals who had undergone assessment for advanced HF therapies (cardiac transplantation/mechanical circulatory support) in either an elective or emergent setting. RHC and CMR are performed as a standard part of the assessment, unless contraindicated, and may have been performed with inotropic support. Patients from Norwich undergoing evaluation for HF with preserved ejection fraction were recruited to provide a diverse range of LVEF, in addition to the Newcastle patients who had predominantly HF with reduced ejection fraction.

RHC was performed via a 7-F sheath inserted into the right internal jugular vein, following established clinical protocols. During the procedure, cardiac output was quantified using the thermodilution technique. A key focus was placed on oxygen saturation measurements, including SvO_2_, which was obtained from blood samples drawn from the pulmonary artery as part of the standard clinical assessment. CMR was performed once patients were deemed to be able to tolerate the examination. The median time between RHC and CMR was 0 ± 2 days.

#### Validation cohort

In the validation cohort, patients were prospectively recruited from the MATCH HF registry between February 2018 and June 2021. The MATCH HF registry includes patients from hospitals within West Yorkshire, United Kingdom, who had been newly diagnosed with HF on echocardiogram (LVEF <50%) and referred for CMR for diagnostic purposes. Patients were excluded if they had a known history of coronary artery disease (coronary stenosis >70% on angiography, known myocardial infarction, previous percutaneous coronary intervention, or coronary artery bypass grafting) or symptoms of angina. Other exclusion criteria included hypertrophic cardiomyopathy, amyloidosis, congenital heart disease, suspected acute pathology, such as myocarditis and advanced renal failure. Patients provided written informed consent. Eligible patients proceeded to CMR and a same-day clinical assessment, including a full clinical review of comorbidities, HF symptoms (NYHA functional class), regular medications, and cardiovascular risk factors. This data were collected in person and via interrogation of electronic patient health records. Patients were followed up for 2 endpoints: HF hospitalization and all-cause death. All patient outcomes were acquired via review of electronic patient records, contact with general practitioners and local coroner’s office where needed.

### CMR study

Patients underwent CMR using a Siemens Sola 1.5-T system in Norwich and Newcastle or a Siemens Prisma-3T system in Leeds (Siemens Healthineers). The imaging protocol for all studies encompassed a minimum of cine imaging for functional assessment and T2 mapping. CMR analysis was independent and blinded to RHC or clinical outcome in the validation cohort.

T2 maps were specifically obtained in the basal short-axis slice of the heart, ensuring uniformity in the region of interest across all subjects. T2 maps were acquired using a standard T2-prepared spoiled gradient echo (FLASH) sequence, with T2 preparation pulses applied at durations of 0, 35, and 55 milliseconds. These durations generated images with distinct T2 weightings, from which T2 relaxation times were obtained. The CMR studies were analyzed using Cvi42 software (Circle Cardiovascular Imaging). Biventricular volumes were calculated by the summation of the discs method.

For T2 mapping, LV and RV blood pools were manually segmented in the basal ventricular short-axis slice, which was found to have less trabeculation in the RV than other slices. Careful attention was given to delineating the endocardial myocardial border and avoiding trabeculation. The RV basal T2 blood pool measurement was labeled RV-T2_BP,_ and the LV basal T2 blood pool measurement was labeled LV-T2_BP_.

We chose to estimate SvO_2_ noninvasively using the ratio of T2 relaxation times of the RV blood pool to the LV blood pool (RV-T2_BP_/LV-T2_BP_). This approach leverages the established sensitivity of T2 relaxation to blood oxygenation levels, where deoxygenated hemoglobin, a paramagnetic agent, shortens T2 in the venous blood of the right ventricle, whereas oxygenated hemoglobin in the arterial blood of the left ventricle results in a longer T2. Consequently, the RV-T2_BP_/LV-T2_BP_ ratio effectively captures the oxygenation gradient across the heart, serving as a robust surrogate for SvO_2_. By normalizing the RV-T2_BP_/LV-T2_BP_, which acts as an internal reference for fully oxygenated blood, this ratio-metric method mitigates the influence of patient-specific factors, such as hematocrit and magnetic field inhomogeneities, that could confound absolute T2 measurements.

### Statistical analysis

All clinically acquired data were assumed to follow a normal distribution. Continuous variables are presented as mean ± SD, whereas categorical data are reported as frequencies and percentages. To account for heterogeneity between groups, continuous variables were compared using a two-sample independent t-test and categorical data were analyzed using the chi-square test. In the discovery cohort, the physiologically relevant ratio of RV-T2_BP_/LV-T2_BP_ was examined for its nonconstant linear association with RHC SvO_2_. This method was selected to ensure the overall equation remained simple and translatable. Further details of the derivation cohort sensitivity analyses are provided in the Supplemental Methods. The CMR iSvO_2_ model was subsequently validated in the external validation cohort to evaluate its clinical relevance to a primary endpoint of death or HF hospitalization. Multivariate prognosis analysis was performed using the Cox proportional hazards model. Statistical analysis was conducted using SPSS (version 23, IBM) and confirmed with MedCalc (MedCalc Software; version 19.1.5). All statistical tests were 2-tailed unless otherwise specified, with a *P* value of <0.05 was considered significant.

## Results

### Study population

Of the 30 patients recruited in the discovery cohort (87% from Newcastle and 13% from Norwich), the mean age was 41 ± 28 years and 67 percent were males (20/30). Key clinical features are shown in Table 1 and include a history of hypertension (7/30, 23%), diabetes (3/30, 10%), and prior myocardial infarction (5/30, 17%). Invasive RHC showed a mean pulmonary artery pressure of 26 ± 11 mm Hg and a cardiac index of 2.3 ± 1.9 L/min. CMR measured left and RV blood pool T2 were 178 ± 34 ms and 104 ± 21 ms, respectively. The validation cohort (n = 628) was older (63 ± 13 vs 41 ± 28 years, *P* < 0.01) with a higher prevalence of hypertension (45% vs 23%, *P* = 0.03). They also showed significantly larger LV end-diastolic volume, LV stroke volume, LVEF, LV mass, RV end-diastolic volume, RV stroke volume, and right ventricular ejection fraction (all *P* < 0.01), whereas RV end-systolic volume was lower (*P* = 0.03) (See Supplemental Table 1 for the full discovery vs validation cohort comparison). Detailed characteristics of the validation cohort are shown in Table 2.

**Table 1:** Discovery Cohort Patient Characteristics (N = 30)

Male	20 (67%)
Age (y)	41 ± 28
Hypertension	7 (23%)
Diabetes mellitus	3 (10%)
Prior myocardial infarction	5 (17%)
Sodium (mmol/L)	132 ± 25
Creatinine (μmol/L)	92 ± 27
Estimated glomerular filtration rate (mL/min/1.73 m^2^)	65 ± 14
Bilirubin (μmol/L)	14 ± 7
Hemoglobin (g/L)	138 ± 20
Hematocrit (%)	0.42 ± 0.05
Mean corpuscular volume (fL)	92 ± 5
Invasive right heart catheter assessment	
Right atrial pressure (mm Hg)	8 ± 5
Pulmonary artery pressure (mm Hg)	30 ± 11
Systolic pulmonary artery pressure (mm Hg)	38 ± 16
Diastolic pulmonary artery pressure (mm Hg)	17 ± 8
Mean pulmonary artery pressure (mm Hg)	26 ± 11
Pulmonary capillary wedge pressure (mm Hg)	17 ± 8
Transpulmonary gradient (mm Hg)	10 ± 6
Cardiac output (L/min)	4 ± 1
Cardiac index (L/min/m^2^)	2.3 ± 1.9
Mixed venous saturation (%)	58 ± 10
CMR assessment	
Left ventricular end-diastolic volume (mL)	168 ± 116
End-systolic volume (mL)	130 ± 97
Left ventricular stroke volume (mL)	45 ± 56
Left ventricular ejection fraction (%)	22 ± 11
Left ventricular mass (g)	90 ± 50
Right ventricular end-diastolic volume (mL)	135 ± 65
Right ventricular end-systolic volume (mL)	101 ± 52
Right ventricular stroke volume (mL)	35 ± 22
Right ventricular ejection fraction (%)	27 ± 12
Heart rate (bpm)	91 ± 18
Left ventricle blood pool T2 (ms)	178 ± 34
Right ventricle blood pool T2 (ms)	104 ± 21

Values are n (%) or mean ± SD.

CMR **=** cardiac magnetic resonance.

**Table 2:** The Validation Cohort (N = 628)

	Death or HF Hospitalization (n = 109)	No Death or HF Hospitalization (n = 519)
Age (y)	67 ± 13	62 ± 13
Male	83 (76%)	329 (63%)
Height (cm)	169 ± 11	169 ± 12
Weight (kg)	82 ± 20	85 ± 20
Resting heart rate (beats/min)	73 ± 15	70 ± 15
Diabetes	25 (23%)	83 (16%)
Hypertension	52 (48%)	231 (45%)
Hypercholesterolemia	32 (29%)	155 (30%)
Cerebrovascular accident	17 (16%)	43 (8%)
Atrial fibrillation	36 (33%)	171 (33%)
Current smoking	22 (20%)	88 (17%)
Past smoking	49 (45%)	196 (38%)
Left bundle branch block	27 (25%)	105 (20%)
Orthopnea	22 (20%)	74 (14%)
Edema	24 (22%)	69 (13%)
NYHA functional class I	47 (43%)	279 (54%)
NYHA functional class II	52 (48%)	197 (38%)
NYHA functional class III	10 (9%)	43 (8%)
Hematocrit (%)	44 ± 6	45 ± 5
Hemoglobin A1C (%)	45 ± 17	43 ± 11
NT-proBNP (pg/mL)	2,166 ± 3514	1,181 ± 2,036
Antiplatelet therapy	31 (28%)	83 (16%)
Beta-blocker therapy	94 (86%)	433 (83%)
Statin therapy	59 (54%)	206 (40%)
Angiotensin-converting enzyme inhibitor/angiotensin II receptor blocker	87 (80%)	387 (75%)
Sacubitril/valsartan therapy	13 (12%)	80 (15%)
Mineralocorticoid receptor antagonist	44 (40%)	227 (44%)
Diuretic therapy	60 (55%)	214 (41%)
Anticoagulant therapy	31 (28%)	166 (32%)
Sodium-glucose cotransporter-2 inhibitor	11 (10%)	106 (20%)
Left ventricular end-diastolic volume (mL)	231 ± 87	215 ± 75
Left ventricular end-systolic volume (mL)	153 ± 85	135 ± 69
Left ventricular stroke volume (mL)	78 ± 25	80 ± 27
Left ventricular ejection fraction (%)	37 ± 13	40 ± 13
Left ventricular mass (g)	142 ± 47	133 ± 43
Right ventricular end-diastolic volume (mL)	165 ± 70	157 ± 49
Right ventricular end-systolic volume (mL)	92 ± 58	82 ± 39
Right ventricular stroke volume (mL)	73 ± 30	75 ± 24
Right ventricular ejection fraction (%)	46 ± 17	49 ± 12
Left atrial volume (mL)	97 ± 50	88 ± 42
Myocardial T2 mapping (ms)	42 ± 4	42 ± 4
Left ventricular blood pool T2 (ms)	124 ± 19	125 ± 20
Right ventricular blood pool T2 (ms)	66 ± 12	71 ± 11
iSvO_2_ (%)	51 ± 11	55 ± 11
Myocardial native T1 (ms)	1,342 ± 59	1,324 ± 50

Values are mean ± SD or n (%).

HF = heart failure; iSvO_2_ = imaging-derived mixed venous oxygen saturation; NT-proBNP = N-terminal pro-B-type natriuretic peptide.

### CMR model of iSvO_2_

**Figure 1:**
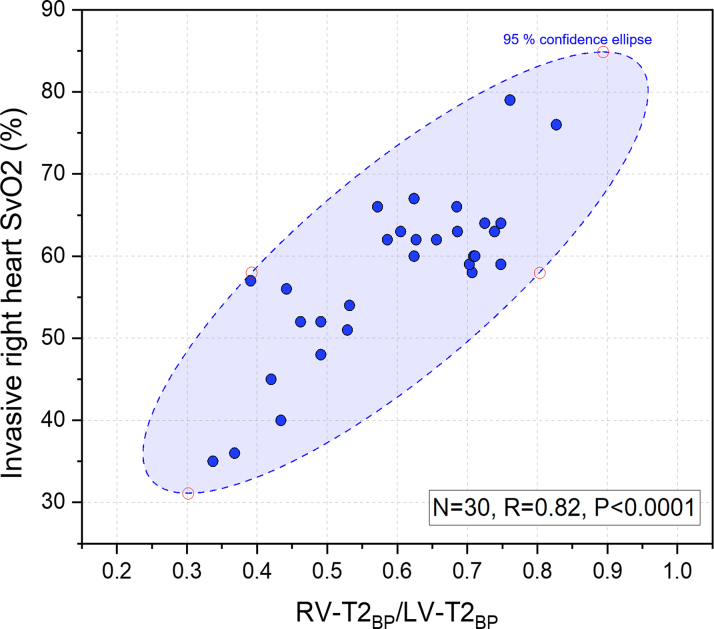
Correlation Between Invasively Measured Right Heart Mixed Venous Oxygen Saturation (%) and RV-T2_BP_/LV-T2_BP_ In the derivation cohort (N = 30) there was a strong positive correlation (R = 0.82, line of best fit [y = 0.95x], *P* < 0.0001) between invasive and CMR derived measurements. LV = left ventricular; RV = right ventricular; SvO_2_ = mixed venous oxygen saturation; T2_BP_ = T2 relaxation time in the blood pool.

A significant positive correlation (R = 0.82; 95% CI: 0.66-0.91; *P* < 0.001) was found between the RV-T2_BP_/LV-T2_BP_ ratio and invasive right heart SvO_2_ levels (Figure 1). A nonconstant linear equation for estimating SvO_2_ from T2 mapping was developed:

$${\text{iSvO}}_{2}=95\cdot \left(\text{RV}-T2_{\text{BP}}/\text{LV}-T2_{\text{BP}}\right)$$

**Figure 2:**
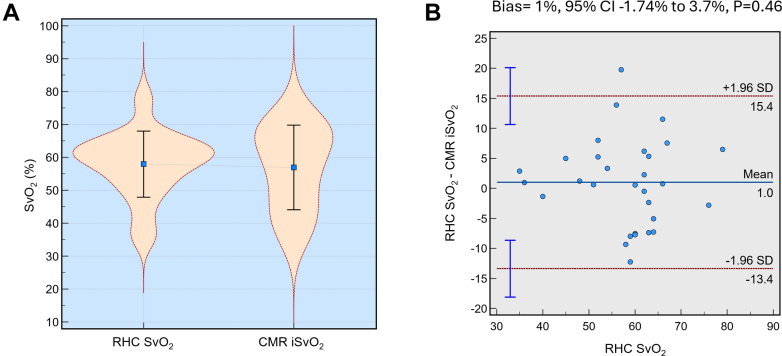
Comparison of Right Heart Catheterization–Measured Mixed Venous Oxygen Saturation and Cardiac Magnetic Resonance–Derived Imaging-Derived Mixed Venous Oxygen Saturation in Heart Failure Patients (A) Violin plots show similar distributions of SvO_2_ for RHC and CMR iSvO_2_ with mean values indicated. (B) Bland-Altman plot showing minimal bias (1%, 95% CI: −1.74% to 3.7%; *P* = 0.46) between RHC SvO_2_ and CMR iSvO_2_, with limits of agreement at ±1.96 SD. CMR = cardiac magnetic resonance; iSvO_2_ = imaging-derived mixed venous oxygen saturation; RHC = right heart catheterization.

Where iSvO_2_ is imaging-derived mixed venous saturation from CMR, RV-T2_BP_ is the RV cavity blood T2 relaxation time, and LV-T2_BP_ is the LV cavity blood T2 relaxation time. There was close agreement between invasive RHC SvO_2_ measurements and those estimated noninvasively using CMR, with a mean bias of 1% and a 95% CI ranging from −1.74% to 3.7% (*P* = 0.46), indicating no significant difference (Figure 2). The limits of agreement were approximately ±15%, suggesting that CMR-derived iSvO_2_ closely approximated invasive SvO_2_ values within an acceptable range. Derivation-cohort sensitivity tests indicate minimal fixed bias (∼1 U), moderate dispersion (coefficient of variability ≈9%), and no meaningful systematic shift, supporting the robustness of agreement between RHC mixed-venous SvO_2_ and CMR iSvO_2_. A comprehensive presentation of the sensitivity analyses used to ensure the robustness of the agreement is provided in the Supplemental Results. In the derivation cohort, estimated SvO_2_ correlated moderately with hemoglobin and hematocrit (r = 0.56 and r = 0.53, respectively) (complete correlation data are provided in Supplemental Table 3).

### Prognostic potential of iSvO_2_ in the validation cohort

**Figure 3:**
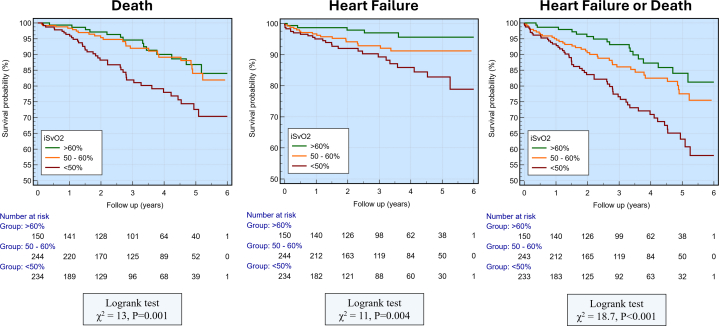
Association of Imaging-Derived Mixed Venous Oxygen Saturation and Clinical Endpoints Kaplan-Meier curves showing the association between iSvO_2_ and the composite endpoint of heart failure or death, death individually, and heart failure individually. (Supplemental Figure 2). Abbreviations as in Figure 2.

Patients were followed up for a median of 3 years (IQR: 1.5-4.8). During the follow-up, 109 (17%) patients fulfilled the primary endpoint criteria of death or hospitalization for HF. Individually, 76 (12%) patients suffered death and 47 (7.5%) hospitalizations for HF. Fourteen patients had both outcomes of HF and death. In those who had met the primary endpoint of death or HF hospitalization, iSvO_2_ was significantly lower (55 ± 11 vs 51 ± 11, *P* = 0.004). Kaplan-Meier curves showing event-free survival for the composite endpoint (death or heart-failure hospitalization) and for the individual endpoints stratified by iSvO_2_ category are shown in Figure 3. Kaplan-Meier subgroup analyses stratified by LVEF (≤40% vs >40%) are shown in Supplemental Figure 1 and are summarized in the Supplemental Results.

In Cox proportional hazard modeling, a significant association was found between iSvO_2_ (per 10% change) and the composite endpoint of death or HF hospitalization (HR: 0.66; 95% CI: 0.54 to 0.81; *P* < 0.001). There was also a significant association between iSvO_2_ and death (HR: 0.72; 95% CI: 0.57-0.90; *P* = 0.004), and HF hospitalization (HR: 0.50; 95% CI: 0.36-0.70; *P* = 0.001) individually (Table 3). Univariate Cox regression results for all evaluated covariates are shown in Supplemental Table 2.

**Table 3:** Cox Regression Derived HRs for Death, Heart Failure Hospitalization, and Composite of Death and Heart Failure Hospitalization

Predictor	Death	Heart Failure Hospitalization	Death or Heart Failure Hospitalization
iSvO_2_ (10% change)			
Unadjusted			
HR (95% CI)	0.72 (0.57-0.90)	0.50 (0.36-0.70)	0.66 (0.54-0.81)
*P* value	0.004	0.001	<0.001
Adjusted for age, sex, and LVEF			
HR (95% CI)	0.76 (0.59-0.98)	0.60 (0.42-0.86)	0.74 (0.60-0.93)
*P* value	0.04	0.005	0.008
Adjusted for age, sex, LVEF, RVEF, NT-proBNP, NYHA functional class, and diabetes status			
HR (95% CI)	0.86 (0.67-1.1)	0.67 (0.44-0.93)	0.78 (0.63-0.98)
*P* value	0.23	0.02	0.04

LVEF = left ventricular ejection fraction; RVEF = right ventricular ejection fraction.

After adjustment for age, sex, and LVEF, iSvO_2_ was still associated with the composite endpoint of death or HF hospitalization (HR: 0.74; 95% CI: 0.60-0.93; *P* = 0.008). Associations between iSvO_2_ and death (HR: 0.76; 95% CI: 0.59-0.98; *P* = 0.04) and HF hospitalization (HR: 0.60; 95% CI: 0.42-0.86; *P* = 0.005) individually remained significant (Central Illustration).

**Central Illustration:**
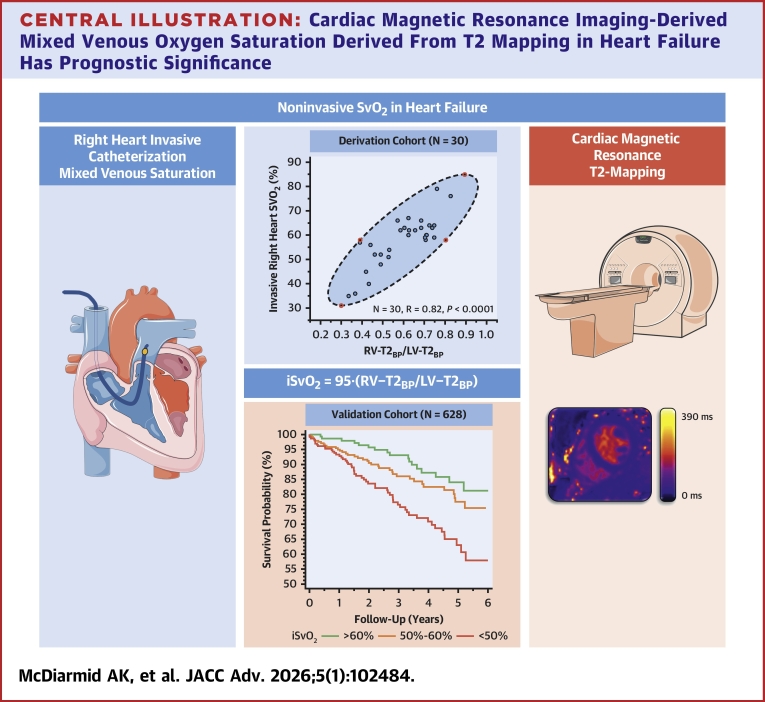
Cardiac Magnetic Resonance Imaging-Derived Mixed Venous Oxygen Saturation Derived From T2 Mapping in Heart Failure Has Prognostic Significance iSvO_2_ = imaging-derived mixed venous oxygen saturation; LV-T2_BP_ = left ventricular blood pool T2 value; RV-T2_BP_ = right ventricular blood pool T2 value; SvO_2_ = mixed venous oxygen saturation.

In a model that included iSvO_2_, age, sex, LVEF, right ventricular ejection fraction, N-terminal pro–B-type natriuretic peptide (NT-proBNP), NYHA functional class, and diabetes status, the association between iSvO_2_ and the composite endpoint of death or HF hospitalization remained significant (HR: 0.78; 95% CI: 0.63-0.98; *P* = 0.04). In this model, iSvO_2_ still had an association with HF hospitalization (HR: 0.67; 95% CI: 0.44-0.93; *P* = 0.02) but no longer had a significant association with death (HR: 0.86; 95% CI: 0.67-1.10; *P* = 0.23).

## Discussion

In this study, we have demonstrated that LV and RV blood pool T2 on CMR can be used to accurately derive pulmonary artery SvO_2_, a measure we have termed iSvO_2_. When applied to a real-world HF registry, iSvO_2_ is a better predictor of outcome than either LVEF or NT-proBNP, and as such may offer an opportunity to derive important prognostic data with CMR that had only previously been possible invasively. Patients with lower iSvO_2_ were typically older, more frequently male, had higher NT-proBNP and an increased prevalence of atrial fibrillation. They also have more severe impairment of both left and right ejection fractions, and a greater indexed LV end-diastolic volume. However, even after correcting for important factors (age, NT-proBNP, and LVEF), iSvO_2_ was still significantly associated with a combined endpoint of death or HF hospitalization.

SvO_2_ is acknowledged as an important tool in the assessment of HF; however, it is not accessible to many patients and clinicians due to a lack of access to invasive RHC. This study has demonstrated that noninvasive measurement of SvO_2_ is possible and could be applied as part of a comprehensive noninvasive HF CMR evaluation.

### Personalized risk stratification

It is known that invasively measured mixed venous saturation of <60% in patients with HF is a marker of adverse outcomes.^24^ Our findings have replicated this noninvasively with iSvO_2_ being predictive of a composite of HF hospitalization and death, even after adjustment for age, sex, LVEF, NT-proBNP, and NYHA functional class.

We have demonstrated that low iSvO_2_ better predicts deterioration in HF independently of LVEF; as such, it may be that it is a more sensitive tool to detect those at risk of progressive pump failure. Other settings where a pattern understanding of the impact of cardiac disease exists, such as predicting arrhythmia, should be studied further.

There may be a role for iSvO_2_ as a gatekeeper to HF interventions, such as implantable cardio-defibrillators. It is not recommended that patients with NYHA functional class IV HF or a prognosis of <1 year receive an implantable cardioverter defibrillator. Despite the validation cohort being composed predominantly of NYHA functional class I and II patients, iSvO_2_ was an independent predictor of admission. It is possible that including iSvO_2_ into cardioverter defibrillator decision-making may avoid implanting defibrillators into high-risk patients and be a more objective marker of risk than a subjective assessment of symptoms.

Furthermore, the novel noninvasive measure we have developed and validated may help identify high-risk individuals and allow for targeted therapies and interventions in an outpatient setting with readily available tools. In advanced HF, younger patients may appear to be relatively well despite a progressive fall in cardiac performance, with the deterioration only being evident once compensatory mechanisms have been exhausted. Implementing routine iSvO_2_ measurement in the longer-term follow-up of this population may allow intervention before clinical deterioration.

### Patient factors and communication

Understanding prognosis is essential in HF, both to guide clinician decision-making and to ensure patients are aware of their disease course.^2^^,^^25^ Living with HF is known to be associated with depression and anxiety.^26^ Improved prognostic tools to better inform discussions with patients are essential if we are to change the mental health burden associated with HF and cardiovascular diseases. The clinical use of iSvO_2_ may provide improved prognostic evaluation for patient groups that would otherwise not undergo advanced HF evaluation with RHC, but for whom CMR may be the standard of care.

Measurement of iSvO_2_ offers an opportunity to noninvasively assess cardiac output and identify cardiovascular risk. RHC is still recommended in the evaluation of patients with HF, although its current use is mainly in those undergoing mechanical assist device and transplantation assessment.^5^ RHC is uncomfortable for patients, expensive, and even in experienced centers, the complication rate of RHC is around 1% with a procedural mortality of 0.06%.^8^ The ability to estimate mixed venous saturation noninvasively by iSvO_2_ using CMR is therefore appealing as it mitigates this risk.

### T2 mapping in clinical practice

It has previously been demonstrated that the oxygen saturation of the blood pool can be estimated using a pulse sequence that incorporates multiple T2 measurements with varying T180 values, obtained with different interecho pulse spacings.^21^ Data from these measurements are processed with oxygen saturation in the Luz-Meiboom model. This method has been validated in a porcine model of progressive hypoxemia. Although this method exhibits excellent correlation with invasive saturation (R^2^ = 0.89), manipulating T180 is not possible on routine clinical CMR scanners, and the calculation is complex. The formula for iSvO_2_, which corrects RV T2 to LV T2, rectifies many of the inherent factors while still yielding a strong correlation (R^2^ = 0.82). The T2 mapping sequence is short (typically only 5-10 seconds for a single slice) and widely available on many scanners, enhancing the generalizability of iSvO_2_. T2 mapping is an established technique^27^ and is shown to only vary to a small degree between different vendors and field strengths. It does not require administration of contrast agents and can be performed in patients with chronic kidney disease. Most T2 mapping sequences used in clinical practice employ single-shot imaging and motion correction, which allows for good results in patients with arrhythmia (one-third of patients in the validation cohort were in atrial fibrillation).

Patients in both our discovery and validation cohorts had to be hemodynamically stable enough to tolerate a 45-minute supine CMR protocol, and our findings do not extend to hemodynamically unstable patients. We envisage that iSvO_2_ will primarily have a role in the management of outpatients or ambulant inpatients with HF. Late referral for advanced therapies, such as an assist device or transplant, leads to poor outcomes, and this technique may allow better identification of patients at high risk of adverse events despite other seemingly reassuring clinical features. Given that T2 mapping is highly reproducible between studies, iSvO2 could potentially be used serially to assess response to treatment.^28^ Future studies are needed to establish how iSvO_2_ responds to acute changes in cardiac output or oxygen delivery.

### Study Limitations

We have not measured arterial saturation in the discovery cohort, and our formula for iSvO_2_ assumes that arterial oxygen saturation is constant. It is therefore unlikely to be accurate in patients with low arterial oxygen saturation, such as those with advanced pulmonary disease. There were no patients with severe anemia in either cohort (as demonstrated by the hematocrit), and given that blood T2 is influenced by hematocrit, iSvO_2_ may be inaccurate in patients with severe anemia. The derivation cohort only included a few patients with very high or low invasive SvO_2_, and the accuracy of iSvO_2_ may be reduced in such cases.

Patients with HF with preserved ejection fraction were not specifically recruited and given the difference in pathophysiology compared with HF with reduced ejection fraction, require further study. Patients in the validation cohort were recruited in the chronic outpatient setting. They may not, therefore, be representative of acute HF presentation, particularly where pulmonary edema affects arterial oxygen saturations.

A single T2 mapping technique has been used in this study, including 2 scanner models (from the same vendor) at 3 centers and at the 2 most clinically used field strengths of 1.5-T and 3.0-T. Further study would be required to evaluate the technique across other T2 measurement methods (eg, MESE or GraSE), vendors, or field strengths to elucidate whether the same iSvO_2_ equation can be used or whether implementation-specific models are required.

## Conclusions

From T2 maps of LV and RV blood pool, it is possible to infer SvO_2_, iSvO_2_, accurately. In a real-world HF registry, iSvO_2_ is a predictor of outcomes and is independent of conventional functional or clinical assessment of HF severity. CMR-measured iSvO_2_ can be measured on existing clinical scanners without the need for additional hardware or sequences.

## Funding support and author disclosures

This research was partly funded by Wellcome Trust, Grant 220703/Z/20/Z and British Heart Foundation FS/CRA22/23034 and supported by National Institute for Health Research Leeds Biomedical Research Centre (NIHR203331). A CC BY or equivalent licence is applied to arising from this submission, in accordance with the grant’s open access conditions. Dr Garg is a clinical advisor for Pie Medical Imaging and Medis Medical Imaging and he consults for Anteris Technologies, Abbott, and Edward Lifesciences. All other authors have reported that they have no relationships relevant to the contents of this paper to disclose.
