# Supplementary material for: Development and Validation of a Noninvasive Model of Mixed Venous Oxygen Saturation in Heart Failure
Source: JACC Adv. 2026 Jan 28;5(1):102484. doi: 10.1016/j.jacadv.2025.102484 (PMC12869880; doi:10.1016/j.jacadv.2025.102484)

Contents

Supplementary Methods 2

Sensitivity analysis in the derivation cohort 2

Sample size calculation 2

Supplementary Results 3

Model Diagnostics 3

Sensitivity analysis 3

Kaplan–Meier survival sub-group analysis results 4

Supplementary TABLES 5

Supplementary Table 1. Comparison of discovery and validation cohort demographics and baseline assessments. 5

Supplementary Table 2. Univariate cox-regression for incidence of HF hospitalisation and death 6

Supplementary Table 3. Correlation of Estimated Mixed Venous Oxygen Saturation (%) with Clinical and Hemodynamic Variables. 9

Supplementary Figure 1. Kaplan–Meier analysis of the composite endpoint of heart-failure hospitalisation or all-cause mortality stratified by indexed mixed venous oxygen saturation ($iSvO_2$). 10

Supplementary Methods

Sensitivity analysis in the derivation cohort

In the derivation cohort (n=30 paired observations), agreement between RHC mixed-venous SVO_2 (reference) and CMR $iSvO_2$ was assessed with a parametric Bland–Altman analysis using the difference (RHC − CMR). We reported the mean difference, the limits of agreement (LoA = mean ±1.96·SD), and 95% confidence intervals for the mean and each LoA. Measurement precision was summarised with the within-subject SD and the corresponding coefficient of variation (CV%). As sensitivity tests for possible systematic shifts, we calculated responsiveness indices—effect size (ES) using the baseline SD and the pooled SD, and the standardised response mean (SRM)—with BCa bootstrap 95% CIs (10,000 iterations). These procedures were used specifically in the derivation cohort to probe the robustness of agreement and bias. Sensitivity to pressure and biochemistry makers.

In the derivation cohort, during catheterisation, mean and systolic pulmonary artery pressures were recorded at end-expiration from high-fidelity waveforms. Pearson correlation coefficients were calculated to explore the influence of anaemia-related variables and pulmonary haemodynamics on T2-derived mixed venous oxygen saturation, with statistical significance predefined as $P < 0.05$ (two-tailed).

No separate protocol was established – all protocol details are in this work.

Sample size calculation

For model derivation, we justify n=30 based on Deng et al., who prospectively included 42 pulmonary hypertension patients and 40 controls and showed the RVT2/LVT2 ratio correlated with oxygen saturations (SVC r=0.564, RA r=0.603, RV r=0.648, PA r=0.582); powering a two-sided α=0.05, 90% test to detect r≥0.60 requires n≈25, so we set n=30 to cover ~20% unusable data (PMID 37060418).

Relevant patient and public involvement was conducted across sites, yielding positive feedback.

All CMR analysis was conducted under the supervision of LEVEL III CMR operators. Any study missing critical results was excluded.

Supplementary Results

Model Diagnostics

A significant positive correlation (R = 0.82, 95% CI 0.66–0.91, $p < 0.001$) was found between the RV-T2BP/LV-T2BP ratio and invasive right-heart SvO_2 (Figure 1). Using ordinary least squares constrained through the origin, we derived a non-constant linear equation to estimate SvO_2 from T2 mapping: $iSvO_2 = 95.26 \times (RV\text{-}T2BP/LV\text{-}T2BP)$ (slope 95.2637 ± 2.2088; 95% CI 90.7462–99.7812; t(29)=43.129; F(1,29)=1860.13; residual SD=7.41; n=30; $p < 0.0001$). The model explained R^2=0.985 of the variance in SvO_2. Residual diagnostics supported model assumptions: residuals were approximately normal (Shapiro–Wilk W=0.9737, p=0.6449), and standard checks did not indicate heteroskedasticity, non-linearity, or dependence.

Sensitivity analysis

The overall mean across measures was 57.4, with a within-subject SD of 5.2 and CV 9.0%. Bland–Altman analysis showed a mean difference of 1.2 units (95% CI −1.6 to 3.9), indicating that CMR $iSvO_2$ was, on average, slightly lower than RHC mixed-venous SVO_2. The LoA were −13.2 to 15.6 units, with 95% CIs of −18.0 to −8.5 (lower) and 10.8 to 20.3 (upper). The reference and CMR means were 58.0 and 56.8, respectively; the pooled SD was 11.5, and the SD of paired differences was 7.3. Responsiveness indices were small and not statistically different from zero: ES (baseline SD) −0.12 (95% CI −0.42 to 0.15), ES (pooled SD) −0.10 (95% CI −0.35 to 0.12), and SRM −0.16 (95% CI −0.52 to 0.22).

Overall, these derivation-cohort sensitivity tests indicate minimal fixed bias (~1 unit), moderate dispersion (CV ≈ 9%), and no meaningful systematic shift, supporting the robustness of agreement between RHC mixed-venous SVO_2 and CMR $iSvO_2$.

Estimated mixed venous oxygen saturation demonstrated significant moderate positive correlations with haemoglobin concentration (r = 0.566, 95% CI 0.22 – 0.78, P = 0.0032) and haematocrit (r = 0.530, 95% CI 0.17 – 0.76, P = 0.0064) (Table 1). No significant associations were identified with serum sodium concentration, serum creatinine concentration, estimated glomerular filtration rate, mean pulmonary arterial

pressure, or systolic pulmonary arterial pressure, each showing weak negative correlation coefficients (r = −0.103 to −0.227) with probability values exceeding the 0.05 threshold.

Kaplan–Meier survival sub-group analysis results

Cumulative survival probability decreased stepwise with lower $iSvO_2$ in both ejection-fraction subgroups. Among participants with LV EF > 40 %, the <50 % $iSvO_2$ category showed the earliest and most pronounced decline, whereas the >60 % category maintained the highest event-free survival throughout follow-up ($\chi^2 = 6.7$, P = 0.03). An analogous pattern was observed in the LV EF ≤ 40 % cohort, where progressive separation of curves was evident and reached statistical significance ($\chi^2 = 7.8$, P = 0.02), with the <50 % group experiencing the greatest cumulative incidence of heart-failure events or death.

Supplementary TABLES.

Supplementary Table 1. Comparison of discovery and validation cohort demographics and baseline assessments.

Variable	Discovery Cohort	Validation cohort	P-value
Number of patients	30	628	
Age (years)	47 ± 14	63 ± 13	<0.01
Male n (%)	20 (69)	412 (66)	0.73
Hypertension n (%)	7 (24)	283 (45)	0.03
Diabetes n (%)	3 (10)	108 (17)	0.33
Left Ventricular End-Diastolic Volume (LVEDV, mL)	168 ± 118	218 ± 77	<0.01
End-Systolic Volume (ESV, mL)	130 ± 98	138 ± 72	0.57
Left Ventricular Stroke Volume (LVSV, mL)	45 ± 57	80 ± 27	<0.01
Left Ventricular Ejection Fraction (LVEF, %)	22 ± 12	39 ± 13	<0.01
Left Ventricular Mass (LVM, g)	90 ± 51	134 ± 44	<0.01
Right Ventricular End-Diastolic Volume (RVEDV, mL)	135 ± 66	158 ± 53	0.02
Right Ventricular End-Systolic Volume (RVESV, mL)	101 ± 53	84 ± 43	0.03
Right Ventricular Stroke Volume (RSV, mL)	35 ± 23	75 ± 25	<0.01
Right Ventricular Ejection Fraction (RVEF, %)	27 ± 12	49 ± 13	<0.01

Supplementary Table 2. Univariate cox-regression for incidence of HF hospitalisation and death

Covariate	beta	SE	Wald	HR	Lower 95%	Higher 95%	P
HF Hospitalisation							
Age (years)	0.04	0.01	9.06	1.04	1.01	1.07	0.003
DM	0.71	0.33	4.68	2.03	1.07	3.84	0.031
HTN	0.22	0.29	0.56	1.24	0.70	2.21	0.454
TIA or CVA	1.04	0.34	9.15	2.84	1.44	5.57	0.003
Smoking	0.35	0.34	1.03	1.42	0.72	2.79	0.310
Hypercholesterolemia	0.20	0.31	0.42	1.23	0.66	2.26	0.516
Oedema	0.79	0.33	5.93	2.21	1.17	4.19	0.015
Orthopnoea	0.49	0.34	2.03	1.63	0.83	3.21	0.154
Diuretic	0.97	0.31	9.88	2.63	1.44	4.81	0.002
Entresto	0.81	0.38	4.51	2.24	1.06	4.73	0.034
ACEI/ARB	-0.11	0.35	0.09	0.90	0.46	1.77	0.759
Beta-blocker	0.76	0.52	2.09	2.13	0.76	5.93	0.148
Anticoagulant	-0.21	0.33	0.41	0.81	0.43	1.54	0.524
Resting HR (bpm)	0.02	0.01	3.81	1.02	1.00	1.04	0.051
NYHA functional class	0.57	0.21	7.40	1.77	1.17	2.68	0.007
NT-proBNP (pg/mL)	0.00	0.00	33.47	1.00	1.00	1.0002	<0.001
LVEF (%)	-0.03	0.01	8.06	0.97	0.95	0.99	0.005
RVEF (%)	-0.02	0.01	5.32	0.98	0.97	0.997	0.021
LA volume (mL)	0.01	0.00	12.07	1.01	1.00	1.01	0.001
Myocardial T2 (ms)	0.02	0.03	0.64	1.03	0.96	1.09	0.424
LV T2 blood pool (ms)	0.00	0.01	0.33	1.00	0.99	1.02	0.565
RV T2 blood pool (ms)	-0.06	0.01	18.71	0.94	0.91	0.97	<0.001

iSvO2 (%)	-0.07	0.02	15.90	0.93	0.91	0.97	<0.001
Death							
Age (years)	0.04	0.01	11.18	1.04	1.01	1.06	0.001
DM	0.43	0.28	2.45	1.54	0.90	2.64	0.118
HTN	0.06	0.23	0.06	1.06	0.67	1.66	0.811
TIA or CVA	0.26	0.34	0.60	1.30	0.67	2.53	0.437
Smoking	0.04	0.30	0.01	1.04	0.58	1.85	0.905
Hypercholesterolemia	0.03	0.26	0.01	1.03	0.62	1.70	0.920
Oedema	0.13	0.30	0.19	1.14	0.63	2.08	0.662
Orthopnoea	0.14	0.30	0.23	1.15	0.65	2.06	0.629
Diuretic	0.27	0.23	1.35	1.31	0.83	2.06	0.246
Entresto	-0.08	0.47	0.03	0.92	0.37	2.32	0.859
ACEI/ARB	0.26	0.32	0.68	1.30	0.70	2.41	0.411
Beta Blocker	0.28	0.34	0.68	1.32	0.68	2.58	0.409
Anticoagulant	-0.04	0.25	0.03	0.96	0.59	1.56	0.863
Resting HR (bpm)	0.01	0.01	0.65	1.01	0.99	1.02	0.421
NYHA functional class	0.39	0.18	4.93	1.48	1.05	2.08	0.026
NT-proBNP (pg/mL)	0.00	0.00	9.39	1.00	1.00	1.0002	0.002
LVEF (%)	-0.01	0.01	0.55	0.99	0.98	1.01	0.458
RVEF (%)	-0.01	0.01	2.14	0.99	0.98	1.00	0.144
LA volume (mL)	0.00	0.00	0.48	1.00	1.00	1.01	0.486
Myocardial T2 (ms)	-0.01	0.03	0.10	0.99	0.93	1.05	0.753
LV T2 blood pool (ms)	0.01	0.01	4.24	1.01	1.00	1.03	0.039
RV T2 blood pool (ms)	-0.02	0.01	3.36	0.98	0.96	1.00	0.067
iSvO2 (%)	-0.03	0.01	8.29	0.97	0.95	0.99	0.004

Abbreviations: ACEI, angiotensin-converting enzyme inhibitor; ARB, angiotensin receptor blocker; bpm, beats per minute; CVA; cerebrovascular accident; DM, diabetes mellitus, HF, heart failure; HTN, hypertension; HR, heart rate; $iSvO_2$, Synthetic mixed venous saturation; LA, left atrium; LV, left ventricle; LVEF, left ventricular ejection fraction; mL; NT-proBNP, N-terminal pro-B-type natriuretic peptide; NYHA, New York Heart Association; RV, right ventricle; RVEF, right ventricular ejection fraction; TIA, transient ischaemic attack.

Supplementary Table 3. Correlation of Estimated Mixed Venous Oxygen Saturation (%) with Clinical and Hemodynamic Variables.

Variable	Correlation coefficient	P-value
Haemoglobin concentration (g dL^{-1})	0.56	0.003
Haematocrit (%)	0.53	0.006
Serum sodium concentration (mmol L^{-1})	–0.22	0.30
Serum creatinine concentration (mg dL^{-1})	–0.13	0.55
Estimated glomerular filtration rate (mL min^{-1} 1.73 m^{-2})	–0.10	0.72
Mean pulmonary arterial pressure (mm Hg)	–0.23	0.28
Systolic pulmonary arterial pressure (mm Hg)	–0.16	0.44

Supplementary Figure 1. Kaplan–Meier analysis of the composite endpoint of heart-failure hospitalisation or all-cause mortality stratified by imaging-derived mixed venous oxygen saturation (iSvO$_2$). Abbreviations: iSvO$_2$, indexed mixed venous oxygen saturation; LV EF, left ventricular ejection fraction.

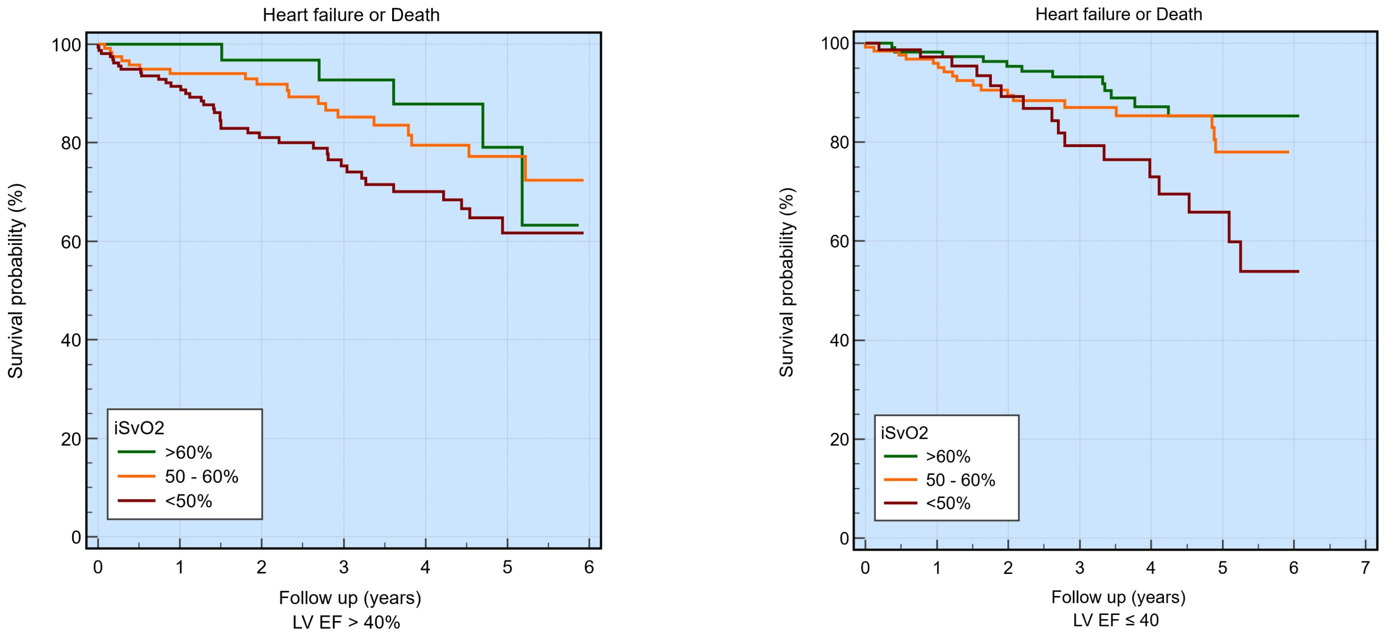

Logrank test
χ^2 = 6.7, P=0.03

Logrank test
χ^2 = 7.8, P=0.02

Supplementary Figure 2. Kaplan-Meier curves with 95% confidence interval for curves from Figure 3.

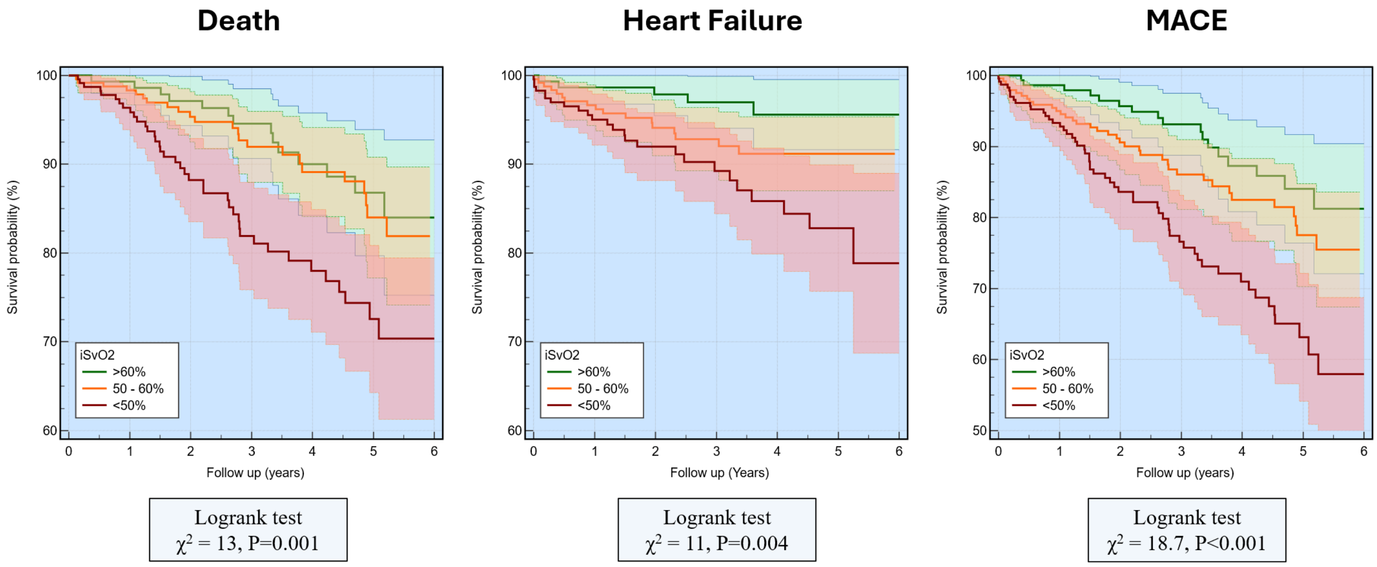

Supplement: Supplementary Material [file mmc1.pdf]
